# Mechanical properties and microstructures data of AISI 1070 steel quenched in epoxidized transesterified cottonseed oil

**DOI:** 10.1016/j.dib.2020.106100

**Published:** 2020-07-30

**Authors:** R.M. Dodo, T. Ause, E.T. Dauda, U. Shehu, J.O. Gaminana, A.P.I. Popoola, E. Mudiare

**Affiliations:** a Department of Metallurgical & Materials Engineering, Ahmadu Bello University, Zaria, Nigeria; b Department of Chemical, Metallurgical and Materials Engineering, Tshwane University of Technology, Pretoria, South Africa

**Keywords:** Epoxides, Transesterified, Quenching performance, Hardness, Strength, Microstructure, ImageJ, Cottonseed oil

## Abstract

The quenching ability of modified and unmodified cottonseed oils was investigated using AISI 1070 steel. In the event of the quenching, the steel samples immersed in each of five distinct quench media, namely epoxidized cottonseed oil (EC), epoxidized-transesterified cottonseed oil (ETC), transesterified cottonseed oil (TC) and fresh cottonseed oil (FC). Tests and analysis conducted determined mechanical properties and microstructures of the quenched samples. The data obtained showed that ETC outperformed other quench media with hardness value of the quenched sample; 407 HVN (hardness value from the FC-quenched sample) increased to 746 HVN indicating an 83.29% improvement. Notably, in the microstructure of ETC-quenched sample, a unique homogeneous microstructure containing a mixture of lath and plate martensite observed with largest martensite per cent of 95.

**Specifications Table**

**Table:** 

**Subject**	**Material science**-Metals and Alloys
**Specific subject area**	Mechanical properties of steels improve by heat treatment processes. Quenching is one of those processes
**Type of data**	Table SEM (Scanning Electron Microscopy) Image Chart Graph Figure
**How data were acquired**	Instruments used include: Ø Instron Universal Testing Machine Ø Vickers hardness machine Ø Avery impact testing machine Ø SEM Ø ImageJ software
**Data format**	Raw analyzed
**Parameters for data collection**	In the quenching operation, austenitizing temperature, austenitizing time and agitation amplitude were the conditions considered for the experiment
**Description of data collection**	description of how these data were collected is given in Experimental Design, Materials and Methods’ section
**Data source location**	Institution: Ahmadu Bello University/ Tshwane University of Technology City/Town/Region: Zaria/Pretoria, Country: Nigeria/ South Africa
**Data accessibility**	The raw and processed data required to reproduce these findings are available with article and available to download from Mendeley Data http://dx.doi.org/10.17632/8cybgmbjvt.1

**Value of the Data**

• Based on the obtained data, all the modified oils (EC, ETC and TC) are able to harden the steel parts with superior as- quenched mechanical properties. However, results reveal that use of ETC outperforms the other modified oils in two respects. First, due to the remarkable thermal-oxidative stability of the ETC, the resultant mechanical properties are outstanding and second, a distinguished microstructure is observed in the parts quenched in the medium
• From the data, ETC can be recommended to be used as fast-oil quenchants in heat treatment industries
• The data expose the possibility of producing quenchants from cottonseed oil with outstanding quenching performance that is completely green (i.e. 100% biodegradable). This will preserve our environment from water pollution and soil contamination normally cause by mineral oil.

## 1 Data description

### 1.1 Mechanical properties data

**Fig. 1:**
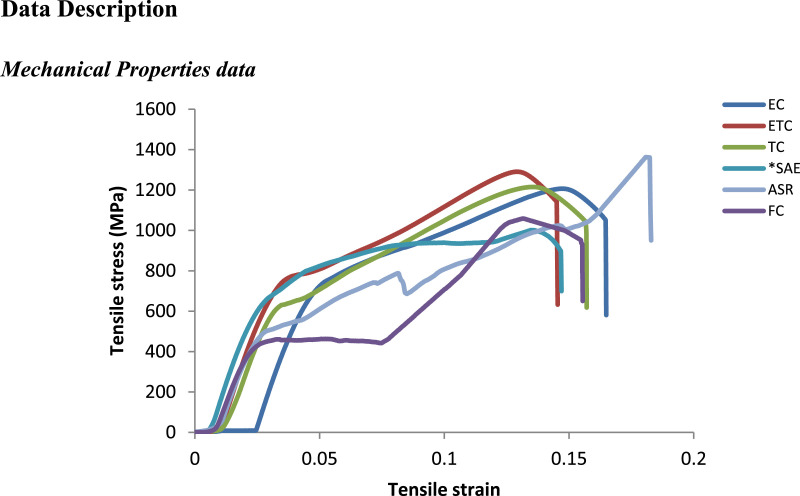
Tensile stress-strain curves for AISI 1070 steel samples quenched in cottonseed oils and SAE40. (*Source: Dodo et al. [1], ASR = sample in as-received condition).

The stress-strain curves registered for the AISI 1070 steel samples quenched in cottonseed oils during tensile test depicted in Fig. 1. It can be inferred that samples quenched in EC, ETC, TC and SAE40 did not yield until then attained the maximum tensile strength of 1206, 1290, 1215 and 1001 MPa respectively. However, FC-quenched and as-received samples yielded at tensile strength of 453 and 784 MPa respectively before reaching the respective tensile stress peak at 1058 and 1362 MPa. Besides, as-received samples yielded before attaining ultimate tensile strength as noted in Fig. 1. ETC quenched samples exhibited superior tensile strengths. These findings are consistent with the report of Technology Products’ Processes [2] that one of the major adjustable parameters in heat treatment that affect the mechanical properties of steels, including the yield strength and hardness is the quench medium. The outstanding quenching performance shown by ETC linked to the marked improvement in the coefficient of heat transfer due to the chemical modification done on the FC. According to many researchers, quenching performance of quenching oils has much to do with how much saturated the oils are [3].

At fracture, as-quenched samples displayed inferior ductility (% of strain) compared to as-received sample. The higher ductility is attributable to the presence of lower bainite structure as viewed in the SEM micrograph obtained for the sample (Fig. 4). Further, it is worth noting that EC and ETC portrayed enormous performance compared with SAE40 due the magnificent thermal-oxidative stability attained. Similar results reported by Otero et al. [4]. The raw data required to reproduce these results are available to download from DOI: 10.17632/8cybgmbjvt.1.

**Fig. 2:**
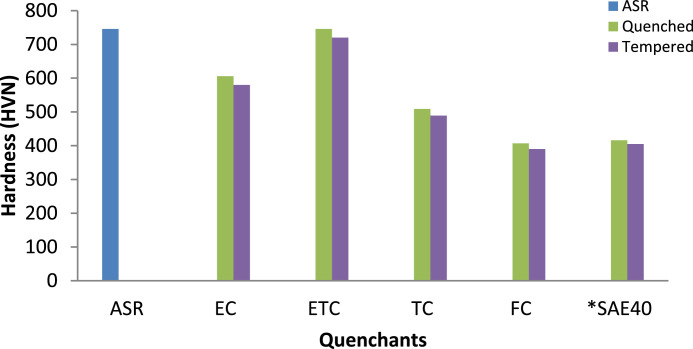
Hardness values for the AISI 1070 steel samples quenched in cottonseed oils and SAE40 (*Source: Dodo et al. [1]).

The data of the hardness values is presented in Figs. 2. The hardness values of the EC, ETC, TC, FC, SAE40 quenched and as-received samples were 606, 746, 509, 407, 416 and 746 HVN respectively. ETC quenched sample produced the highest hardness values followed by samples from EC while FC quenched sample displayed inferior hardness values. The hardness developed by SAE40 is comparable to that from the raw oil. The trend could be explained by the high cooling rate offered by the ETC as a result of the structural modification. However, in a study conducted by Adekunle et al. [5], it was reported that austenitizing temperature significantly influences the developed hardness after quenching in various unmodified vegetable oils. Additionally, from Fig. 2 after tempering treatment, there was a slight drop in hardness. This connected to the structures obtained after tempering which revealed an increased precipitation of stable cementite in fine super-saturated ferrite and lower bainite, due to decomposition of martensite and retained austenite (Fig. 8). This is in line with what Dodo et al. [6] reported. To reproduce the findings, the raw data can be downloaded from DOI: 10.17632/8cybgmbjvt.1.

**Fig. 3:**
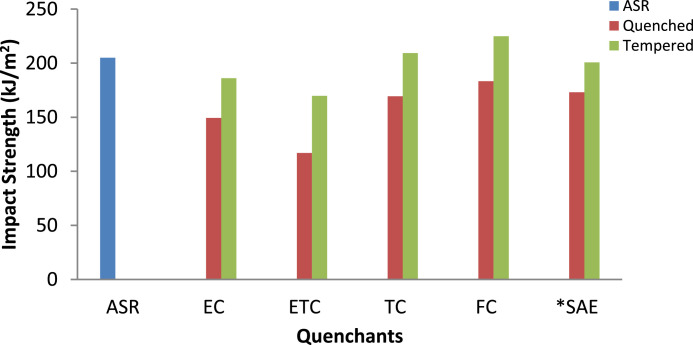
Impact Strength of the AISI 1070 steel samples quenched in cottonseed oils and SAE40 (*Source: Dodo et al. [1]).

Fig. 3 indicate the impact strength (energy absorbed per unit area under the notch) for samples quenched in different media. It is notable that highest impact strength obtained from sample quenched in FC. TC-quenched sample was next to it, followed by sample quenched in EC. Inferior impact strength shown by ETC-quenched sample. This trend could be attributed to the relative amount of the martensite and the retained austenite achieved in the various samples after quenching from each of the quenchant. This is close to the existing literature [7]. Furthermore, the impact strength improved significantly after tempering. This is due to the structure obtained (tempered martensite) alongside with stress-relieve after tempering. The raw data are available to download from DOI: 10.17632/8cybgmbjvt.1 for reproducing.

### 1.2 Microstructural analysis

**Fig. 4:**
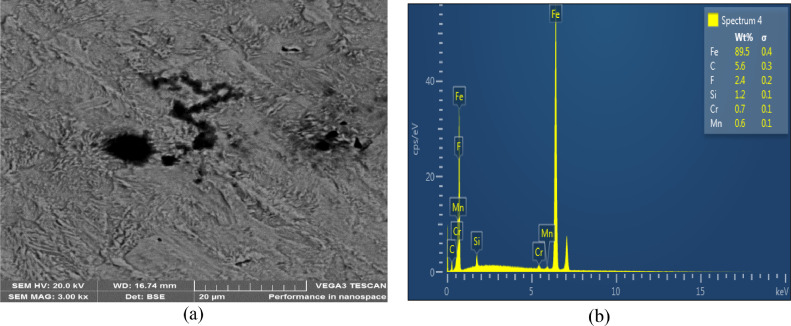
a) SEM Image of as-received sample; b) ED spectrum of the major elements for as-received sample. 2% Nital etch.

**Fig. 5:**
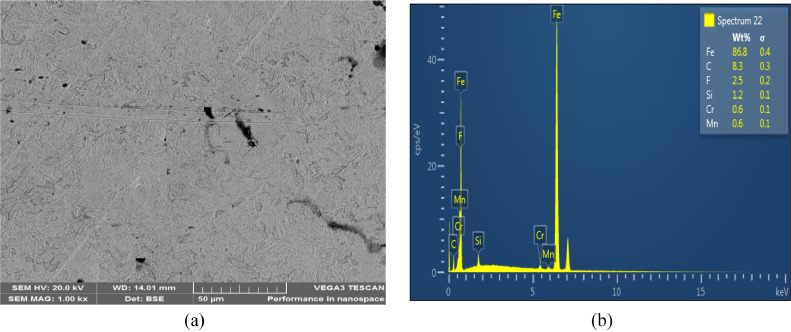
a) SEM Image of samples quenched in SAE40 b) ED spectrum of the major elements. 2% Nital etch *(Source*: Dodo et al. [1]).

**Fig. 6:**
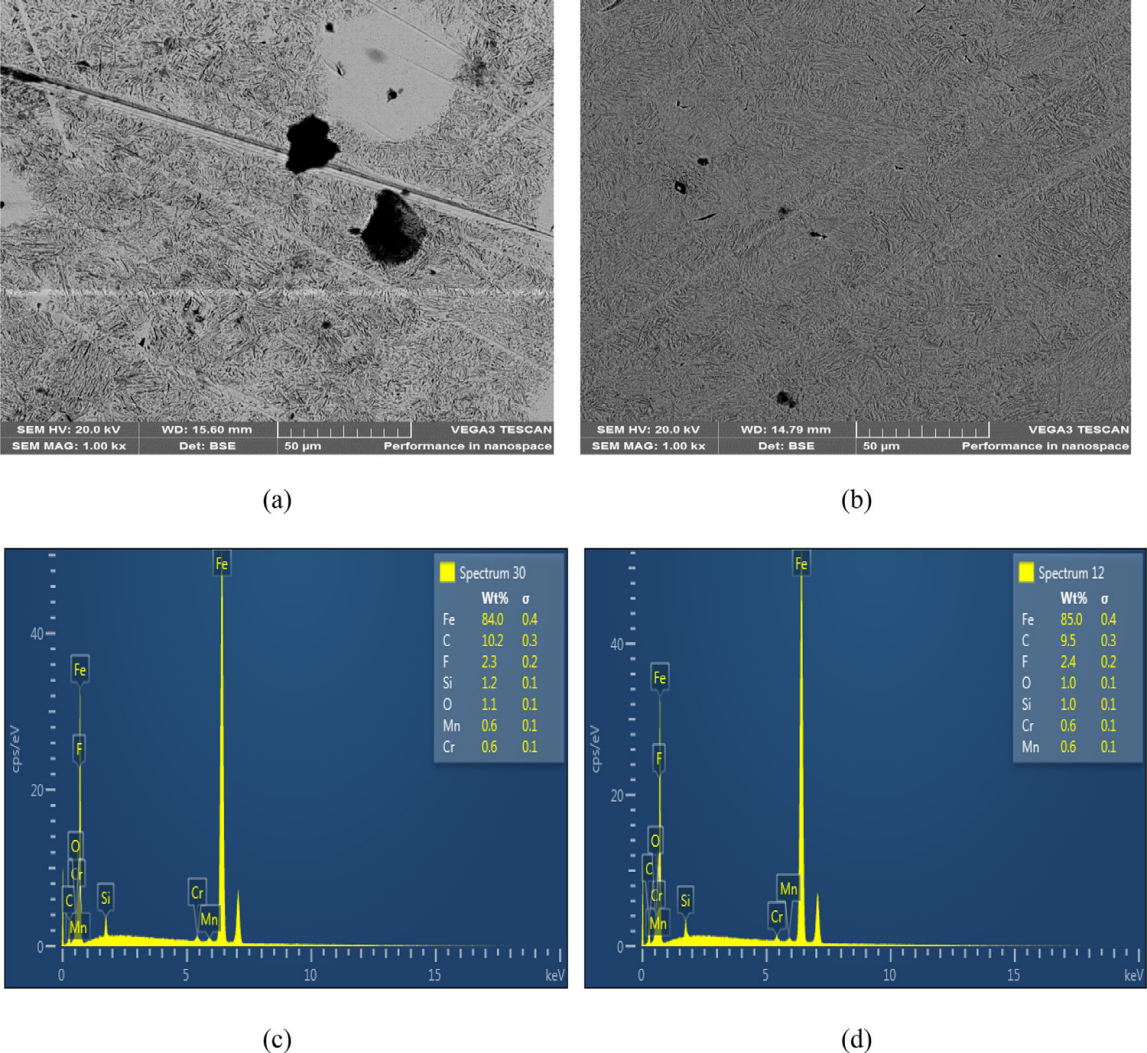
SEM Images of samples quenched in a) EC, b) ETC; ED spectrum of the major elements for samples quenched in c) EC, d) ETC. 2% Nital etch.

**Fig. 7:**
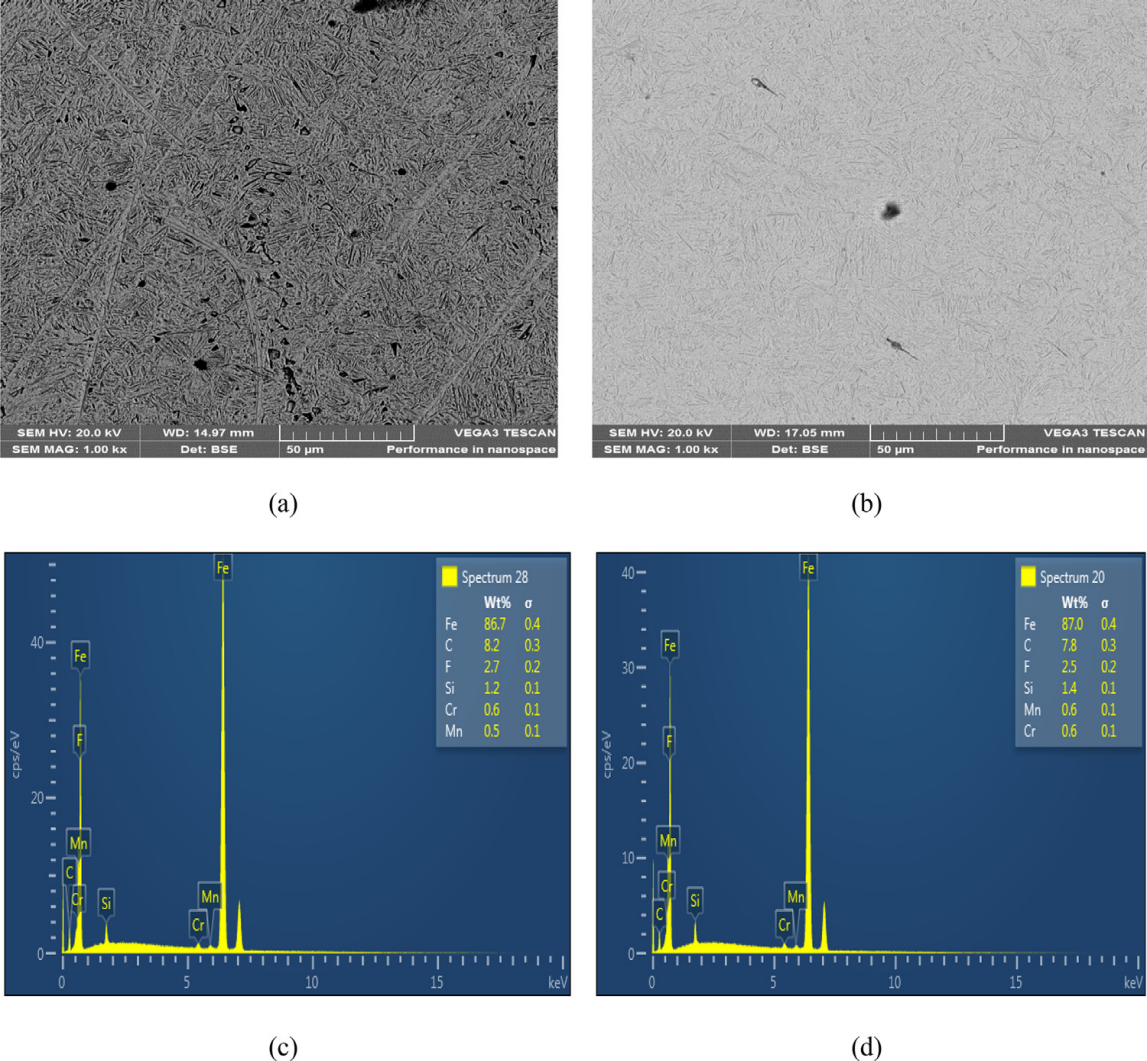
SEM Images of samples quenched in a) TC, b) FC; ED spectrum of the major elements for samples quenched in c) TC, d) FC. 2% Nital etch.

**Fig. 8:**
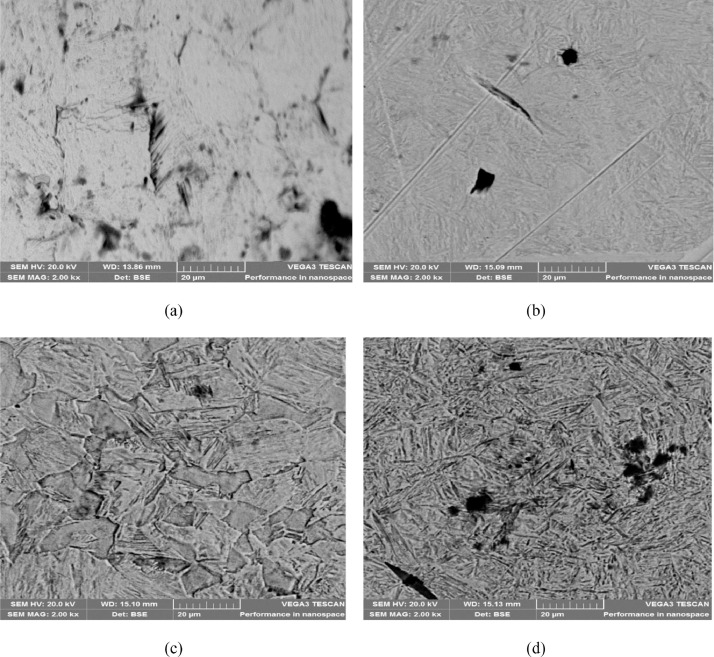
SEM Images of tempered samples after quenching in a) EC, b) ETC, c) TC, d) FC. 2% Nital etch.

The microstructures of the as-received, quenched and tempered samples showed in Fig. 4, Fig. 5, Fig. 6, Fig. 7, Fig. 8. The as-received consists of lower bainite. Martensite and retained austenite is contained in samples quenched in EC, ETC, TC, FC and SAE40. ETC-quenched sample reveals large volume of martensitic structure followed by sample quenched in EC and then TC. The influence of the structure seen in the developed hardness with the sample quenched in ETC reaching the maximum hardness (Fig. 2). Nonetheless, compared to samples quenched in modified oils, SAE40-quenched sample showed a lower martensite content. Possibly, modified oils provide higher cooling rate than SAE40. In similar manner, Vivek et al. [8] observed highest per cent of martensite in brine quenched samples. However, water quenched samples showed intermediate martensite per cent while oil quenched samples showed the least martensite content.

The structure of the tempered samples showed an increase in precipitation of cementite in fine supersaturated ferrite and lower bainite due to the decomposition of martensite and retained austenite (Fig. 8). The decomposition accounted for the slight fall in hardness and strength after tempering. The observations are in line with that of Hassan et al. [9]. For use in future research, the raw data are available to download at DOI: 10.17632/8cybgmbjvt.1.

Table 1 revealed the data of the image analysis conducted using ImageJ software. It is obvious from the Table that sample quenched in ETC have the highest martensite per cent and largest number of lath count. This could be connected to the excellent quenching performance shown by ETC which is a direct consequence of the chemical modification done. Sample with microstructural parameters second to that quenched in ETC was EC-quenched sample. Next to it was TC-quenched sample and then finally FC quenched sample had the inferior microstructural parameters. However, Abbas and Afiaa [10] recorded low martensite per cent after running image analysis using ImageJ software. The difference in the martensite fraction could be attributed to the dual phase steel used in the analysis.

## 2 Experimental design, materials and methods

### 2.1 Modifications process

The chemical structure of FC modified by transesterification and/or epoxidation processes according to the method utilized by Dodo et al. [11] and Dodo et al. [12] respectively.

The first step in the transesterification took place by dissolving 0.5 g of catalyst (NaOH) in 32.6 g of methanol. The mixture was then poured into 100 g of the oil sample and subsequently placed on a magnetic stirrer at 60 °C for stirring (450 rpm) and heating for 1 h. Finally, the solution was poured into a separating funnel for the separation of FAME (fatty acid methyl ester) and glycerol. The denser glycerol was drained. Afterwards, the ester was purified by water washing and then drained [11].

Epoxidation, on the other hand carried out as follows:

0.297 g of H_2_SO_4_ (catalyst) was slowly added in drops to the 4.642 g of acetic acid and then the solution mixed with 40 g of the oil sample. At a steady speed of 450 rpm, the mixture stirred continually; 17.536 g of hydrogen peroxide in 30% solution was added slowly. The mixture heated to 50 °C gradually. Mixing acetic acid with hydrogen peroxide simultaneously generated peroxide acid *in situ*. Lastly, the mixture poured into a separating funnel after 2.5 h and the aqueous layer was immediately drained. With 15 per cent NaOH solution, the product neutralized, washed carefully with hot water and lastly dried by evaporation [12].

### 2.2 Heat treatment methods

#### 2.2.1 Sample preparation

AISI 1070 steel samples were machined to the ASTM standard dimensions [13]. Required volume of EC, ETC, TC and FC (maintaining 10 °C rise in temperature during quenching) were measured and placed in separate containers. Also, SAE40 measured and poured into fifth separate container. SAE40 was the mineral oil used for comparison.

#### 2.2.2 Normalizing

Samples were heated to 800 °C and soaked for 75 min and then air-cooled.

#### 2.2.3 Hardening by quenching

The promising experimental conditions for optimum hardness development for each quench medium reported by Dodo et al. [14] were used. These parametric conditions (Table 2) implemented for each quench medium. Normalized samples austenitized at the required temperature, soaked and then quickly immersed in the agitated EC, ETC, TC, FC and SAE40. There were fourteen samples for each of the five containers. The operation carried out at room temperature of 27 °C. Laboratory Sieve shaker used to give the needed agitation during the operation.

**Table 1:** Data of Image analysis (*Source: Dodo et al. [1]).

	Relative amount of phases present (%)				
Quenchants	Martensite	Retained austenite	Count	Perimeter (µm)	Feret's Diameter (µm)
EC	93	7	580	11.845	2.482
ETC	95	5	675	10.714	2.048
TC	89	11	500	13.845	2.581
FC	84	16	227	18.707	2.990
*SAE40	77	23	252	14.5	2.80

**Table 2:** Heat treatment conditions for optimum hardness *(Source*: Dodo et al. [14]).

Quenchant	Austenitizing Temperature ( °C)	Austenitizing time (min)	Agitation Amplitude (mm)
EC	800	30	3
ETC	850	30	1.5
TC	850	30	1.5
FC	800	45	1.5
SAE40	800	45	3

#### 2.2.4 Tempering

Half of the quenched samples from the quench media tempered. This involved heating the samples to 350 °C and then holding for 40 min. Afterwards, samples air-cooled.

### 2.3 Mechanical properties tests

#### 2.3.1 Tensile test

**Fig. 9:**
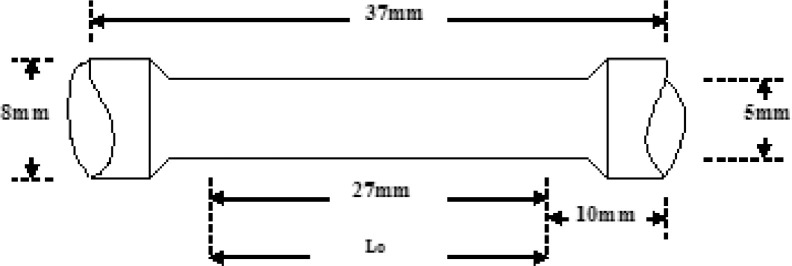
Standard tensile test piece (*Source:* ASTM [13]).

The tests performed using Instron Universal Testing Machine (Instron-3369 series, Corp., Norwood, USA). The load resolution and the minimum displacement rate of the machine used were 0.5 mN and 0.001 mm/min respectively. A constant strain rate of 2.764 × 10^−3^s^−1^ used for all tests samples. The test method followed was ASTM E8. The sample held at its ends on to the grips of the machine. The stress-strain and percentage elongation graphs recorded digitally from the computer connected to the machine. The test sample used depicted in Fig. 9.

#### 2.3.2 Hardness test

The hardness values of the test samples determined using Vickers hardness machine (MV1-PC model, Serial No.: 07/2012–1329). The test performed in accordance of the method reported by Dodo et al. [14]. A diamond indenter was used to indent the surface of the test sample by the application of static load of 0.3kgf, which was maintained for fifteen minutes.

#### 2.3.3 Impact test

The toughness of each sample determined using Avery impact testing machine with maximum capacity of 120 ft.lb (machine no. E 51,425/9, type 6701). The test method used is in line with ASTM E23. The pendulum set to a potential energy position of 162.75 J before the test. The test sample griped vertically and the trigger released and immediately the registering pointer of the quadrant scale marked the energy absorbed in Joules in breaking the sample. The energy absorbed was taken. Impact strength (energy absorbed per unit area under the notch) calculated from the impact energy and the sample dimension. The same repeated for other test samples.

### 2.4 Microstructural analysis

#### 2.4.1 Microstructural examination

Conventional metallographic preparation procedure was used to prepare samples for microstructural analysis. The scanning electron microscope, TESCAN (model: VEGA3) was used to image and examine the phases present.

#### 2.4.2 Image analysis

ImageJ software used to determine relative proportion of martensite and retained austenite present in the quenched samples. The analysis ran using the following steps.

The SEM image opened in ImageJ using “*File → open* → *SEM image”* function. Next, the image converted to greyscale using “*Image* → *Type* → *8-bit”*. Afterwards, scale of measurement set; a line drawn over a 50 µm section of the ruler then “*analyse* → *Set Scale”* function clicked*.* In *Set Scale* window, 50 entered into the 'known distance' box and changed the 'unit of Measurement' box to µm. Subsequently, the image duplicated excluding the scale bar using rectangular selection tool by clicking “*Image* → *duplicate”* function. Then, the image was thresholded (coloured) by “*Image* → adjust → threshold*”* function. This involved sliding the bars to colour red the phase of interest and then clicked apply. Lastly, the relative amount of the martensite (area fraction in ImageJ) measured using *“Analyse → measure”* function. The result was recorded.

Equally, the software used in obtaining the length and the number of the martensitic laths or plates per square micro metre (µm^2^). Appropriately, the following steps were taken.

The first five steps of the above analysis repeated. Laths/plates on the image then outlined by clicking “*process* → *binary → outline”* function. Afterwards, the average length and the number of the laths/plates per square micro metre measured using *“analyse → analyse Particles”*. The particle analyser configured as follows:

0.1-infinity and 1–0.8 assumed to be the size range and the circularity respectively, 'Show Outlines', toggled and then‘Display Results'and‘Summarize’ checked before clicking 'OK’. At the end, results and the summary of the laths/plates analyses presented in separate data windows.

## Declaration of Competing Interest

The authors declare that they have no known competing financial interests or personal relationships which have, or could be perceived to have, influenced the work reported in this article.

## Appendix Supplementary materials

Image, application 1
